# Nitroglycerin versus milrinone for low central venous pressure in patients undergoing laparoscopic hepatectomy: a double-blinded randomized controlled trial

**DOI:** 10.1186/s12871-024-02631-5

**Published:** 2024-07-18

**Authors:** Huayan Lv, Xiaofeng Jiang, Xiaoxia Huang, Wei Wang, Bo Wu, Shian Yu, Zhijian Lan, Lei Zhang, Yuwen Lao, Jun Guo, Na Yang, Na YangNo

**Affiliations:** 1 Department of Anesthesiology, Jinhua Hospital Affiliated to Zhejiang University School of Medicine, Jinhua, Zhejiang Province People’s Republic of China; 2 Department of Hepatological Surgery, Jinhua Hospital Affiliated to Zhejiang University School of Medicine, Jinhua, Zhejiang Province People’s Republic of China; 3 No. 365, Renmin East Road, Wucheng District, Jinhua, Zhejiang Province 321000 People’s Republic of China

**Keywords:** Laparoscopic hepatectomy, Milrinone, Low central venous pressure

## Abstract

**Background:**

Conventional anesthesia used to reduce central venous pressure (CVP) during hepatectomy includes fluid restriction and vasodilator drugs, which can lead to a reduction in blood perfusion in vital organs and may counteract the benefits of low blood loss. In this study, we hypothesized that milrinone is feasible and effective in controlling low CVP (LCVP) during laparoscopic hepatectomy (LH). Compared with conventional anesthesia such as nitroglycerin, milrinone is beneficial in terms of intraoperative blood loss, surgical environment, hemodynamic stability, and patients’ recovery.

**Methods:**

In total, 68 patients undergoing LH under LCVP were randomly divided into the milrinone group (*n* = 34) and the nitroglycerin group (*n* = 34). Milrinone was infused with a loading dose of 10 µg/kg followed by a maintenance dose of 0.2–0.5 µg/kg/min and nitroglycerin was administered at a rate of 0.2–0.5 µg/kg/min until the liver lesions were removed. The characteristics of patients, surgery, intraoperative vital signs, blood loss, the condition of the surgical field, the dosage of norepinephrine, perioperative laboratory data, and postoperative complications were compared between groups. Blood loss during LH was considered the primary outcome.

**Results:**

Blood loss during hepatectomy and total blood loss were significantly lower in the milrinone group compared with those in the nitroglycerin group (*P* < 0.05). Both the nitroglycerin group and milrinone group exerted similar CVP (*P* > 0.05). Nevertheless, the milrinone group had better surgical field grading during liver resection (*P* < 0.05) and also exhibited higher cardiac index and cardiac output during the surgery (*P* < 0.05). Significant differences were also found in terms of fluids administered during hepatectomy, urine volume during hepatectomy, total urine volume, and norepinephrine dosage used in the surgery between the two groups. The two groups showed a similar incidence of postoperative complications (*P* > 0.05).

**Conclusion:**

Our findings indicate that the intraoperative infusion of milrinone can help in maintaining an LCVP and hemodynamic stability during LH while reducing intraoperative blood loss and providing a better surgical field compared with nitroglycerin.

**Trial registration:**

ChiCTR2200056891,first registered on 22/02/2022.

## Background

Laparoscopic hepatectomy (LH) was previously considered unfeasible due to the complex vascular anatomy and the unique risk of bleeding associated with the liver. However, advancements in surgical laparoscopic techniques, equipment, and anesthesia over the past two decades have revealed LH as a safe, minimally invasive, and effective surgical procedure; therefore, it is a widely performed technique [1]. Laparoscopic hepatic hemostasis techniques are more challenging compared with open liver resection. Intraoperative blood loss is a major challenge faced by hepatic surgeons and also a key factor influencing the success of laparoscopic surgery. Blood loss during liver transection can be reduced through various strategies such as temporary vessel occlusion (including inflow occlusion and outflow occlusion); the use of different hemostatic instruments, equipment, and hemostatic topical agents; and the implementation of appropriate anesthesia methods. These anesthesia methods include low central venous pressure (LCVP), hypoventilation, the reverse Trendelenburg’s posture, epidural anesthesia, normovolemic hemodilution, and different pharmacological interventions [2, 3]. LCVP is recommended during hepatic transection by Enhanced Recovery After Surgery (ERAS) Society to minimize the risk of bleeding during surgery (evidence level: high; grade of recommendation: strong) [4]. LCVP is associated with reduced blood loss, length of hospital stay, and surgical time [3, 5].

No standard protocols are currently available on the details of LCVP management, and the best technique to achieve LCVP during liver resection has not been established [4, 6, 7]. Conventional anesthesia used to minimize CVP includes intravenous (IV) fluid restriction during surgery, proper patient positioning, and the use of different drugs such as glyceryl trinitrate and diuretics [6, 7]. Nevertheless, fluid restriction along with intraoperative blood loss and glyceryl trinitrate can lead to hemodynamic instability [5]. In our previous study, we found that hypotension occurs frequently in patients undergoing LH under LCVP. We found that the mean arterial pressure (MAP) decreased by more than 20% in 84.7% of the patients [8]. Ryu et al. [9]. reported that milrinone can provide a superior surgical environment and hemodynamic stability during living donor hepatectomy. A recent study also reported that milrinone is a better choice to reduce CVP with better hemodynamic manifestation and improved postoperative recovery compared with nitroglycerin during hepatectomy [10]. The results are favorable although the study only assessed open hepatectomy; therefore, its effect on patients undergoing LH is not yet established. Additionally, milrinone is clinically less commonly used to reduce CVP. Furthermore, its benefit in reducing CVP and bleeding in the surgical field during LH remains unclear [11], which warrants further investigation.

Theoretically, a vasodilator agent that possesses a positive inotropic effect could potentially provide a more rational approach for controlled LCVP and clinical outcomes during LH compared with a pure vasodilator. We hypothesized that milrinone can be effective in minimizing intraoperative blood loss during LH while also effectively controlling LCVP compared with nitroglycerin. Therefore, in this study, we aimed to compare the efficacy and safety of milrinone and nitroglycerin in controlling LCVP during LH and to assess their effect on clinical outcomes. We established a simple, standardized, and safe approach for LCVP anesthesia during LH.

## Materials and methods

### Study design

This study was approved on 30 January 2022 by The Ethics Committee of Jinhua Hospital Affiliated to Zhejiang University, Zhejiang, China (Protocol Number: 2022–004–001), and the trial was registered at the Chinese Clinical Trial Registry (http://www.chictr.org.cn, ChiCTR2200056891) on 22/02/2022. After receiving approval for clinical registration, the trial was performed from June 2022 to July 2023.

After obtaining informed consent, we enrolled 68 consecutive patients scheduled for LH under LCVP for a minimum of 2 hours at Jinhua Hospital Affiliated to Zhejiang University. The study followed a prospective, randomized, double-blinded design. The inclusion criteria were as follows: patients aged between 18 and 70 years, with American Society of Anesthesiologists (ASA) grade I–II, operated by a single surgeon. The exclusion criteria were as follows: patients with a body mass index (BMI) of more than 35 kg/m^2^; a history of cranial cerebral trauma surgery, stroke, central nervous system diseases, or psychiatric diseases; primary hypertension > 180 mmHg, uncontrolled hypertension, or postural hypotension; severe heart diseases, such as hypertrophic cardiomyopathy, myocardial infarction, NYHA cardiac function grades III–IV, and echocardiographic of LVEF < 50%. Furthermore, the following patients were also excluded: patients with hepatic decompensation, patients with moderate and severe valve regurgitation and preoperative arrhythmia, dyspnea, or respiratory failure; recent (within 2 weeks) use of heparin, antithrombin, warfarin anticoagulant factor, aspirin antiplatelet aggregation, or other anticoagulant drugs; and known allergy to any drug used in the study.

In accordance with the approved study protocol by the Institutional Review Board, patients were randomly assigned to two groups in a 1:1 ratio using a computer-generated randomization table: the milrinone group and the nitroglycerin group, each comprising 34 patients. The randomization sequences were enclosed within sealed opaque envelopes and managed by two nurse anesthetists who were not involved in the eligibility assessment and recruitment of patients. The nurse anesthetists opened the envelopes and administered the experimental drug once the patients were in the operating room. Throughout the trial, patients, surgeons, and the main researcher responsible for patient anesthesia and follow-up survey data collection remained blinded to the group assignments.

### Management of patients

All patients underwent a standardized anesthetic protocol without premedication. In the preoperative preparation room, a peripheral intravenous line was used for all patients by the nurse. Subsequently, internal jugular double-lumen catheterization was performed by the anesthetist in the preparation room under local anesthesia. Fluid administration was restricted to an infusion rate of 2–3 mL/kg/h before anesthesia induction. Upon entering the operating room, with standard monitoring and CVP monitoring (the central venous catheter line was also connected to the transducer), anesthesia was induced using atropine (0.2 mg), sufentanil (0.8 µg/kg), etomidate (0.2–0.3 mg/kg), and cis-atracurium (0.3 mg/kg). Radial arterial puncture and subcostal transversal fascia block were performed by the attending anesthetist after anesthesia induction and stabilization. The arterial line was connected to the Vigileo/FloTrac monitor (Edwards Lifesciences, Irvine, CA, US) for real-time hemodynamic monitoring of arterial pressure, cardiac index (CI), cardiac output (CO), and systemic vascular resistance index (SVRI). Anesthesia was maintained with propofol, remifentanil, cis-atracurium, and sevoflurane (0.5–1%). Mechanical ventilation was started with a fraction of inspired oxygen (FiO_2_) of 50% and a tidal volume of 8 mL/kg at a frequency of 12/min. Minute ventilation was adjusted to maintain the end–tidal carbon dioxide partial pressure (PetCO_2_) between 35 and 50 mmHg. Throughout the surgery, the anesthesiologist regulated the anesthesia to maintain the bispectral index (BIS) of anesthetic depth between 40 and 60.

After inducing anesthesia in both groups, fluid administration was restricted with an infusion rate of 2–3 mL/kg/h before transecting the liver parenchyma. Either milrinone or nitroglycerin was administered once pneumoperitoneum was established. In the milrinone group, 10 µg/kg milrinone was administered over 10 min as a loading dose, followed by its infusion at a rate of 0.2–0.5 µg/kg/min until the removal of the liver lesions. In the nitroglycerin group, nitroglycerin was administered at a rate of 0.2–0.5 µg/kg/min. Either milrinone or nitroglycerin infusion was stopped after complete parenchymal transection. In both groups, performing the intermittent Pringle maneuver to reduce bleeding was at the discretion of the surgeon and was used in most patients. The intermittent Pringle maneuver was performed in cycles of 15/5 min for clamping/unclamping using an appropriately prepared laparoscopic Foley catheter of the hepatic pedicle. The fluid infusion rate during liver parenchymal transection was adjusted by the attending anesthesiologist according to the CVP and surgical field condition. The surgical field condition during liver parenchymal transection was evaluated using a four-point grading scale [9, 12]: grade I is very lax hepatic veins, minimal bleeding at the resection plane, and very easy to operate; grade II is lax hepatic veins, a little bleeding at the resection plane, and easy to operate; grade III is tense hepatic veins, appreciable bleeding at the resection plane, and somewhat difficult to operate; and grade IV is very tense hepatic veins, profuse bleeding at the resection plane, and very difficult to operate. After completing parenchymal transection, the rate of intravenous fluid administration was increased to maintain the CVP close to baseline before induction. Fluids were administered intraoperatively using the lactate Ringer solution routinely, whereas the colloid solution was administered for volume replacement only when blood loss was more than 500 mL. Moreover, packed concentrated red blood cells were transfused if the intraoperative red blood cell volume detected by a blood gas analysis was lower than 25%. The total volume of intraoperative blood loss was the sum of the volumes of blood present in the suction systems and gauzes.

During the surgery, the case report form was manually recorded by an anesthetic assistant according to the device data. The hemodynamic data (heart rate [HR], pulse oximetry (SpO_2_), invasive MAP, CVP, BIS, PetCO_2_, CI, SVRI, and CO) were recorded at the following predefined time points: before (T0) and after induction (T1), before milrinone/nitroglycerin administration (T2), 10 min after milrinone/nitroglycerin administration (T3), right before the initiation of liver parenchymal transection (T4), midway through liver parenchymal transection (T5), right after the completion of parenchymal transection (T6), and at the end of the surgery (T7). Noradrenaline was administered to the patients in the two groups to maintain an MAP of more than 65 mmHg. In both groups, perioperative hypotension was defined as a 30% reduction of MAP from baseline.

Blood samples were collected to identify end-organ perfusion markers (lactate concentration) and examine routine blood factors at the beginning and end of the surgery. After the surgery, the patients woke up, were extubated in the operating room, and then were transferred to the post-anesthesia care unit with an intravenous patient-controlled analgesia device. The blood samples were taken for liver and renal function tests preoperatively, immediately after the surgery, and on postoperative days 1, 3, and 7. The diagnosis of liver cirrhosis was confirmed by the liver computed tomography and pathologic examination of the resected specimen. Additionally, we monitored postoperative recovery and complications. Postoperative complications were classified according to the Clavien–Dindo classification [13]. Postoperative mortality was defined as any death within 30 days after the surgery.

### Study outcomes

The primary outcome was the difference in blood loss between the milrinone group and the nitroglycerin group during the surgery. The secondary outcomes included the surgical field condition during liver resection, hemodynamic stability, fluid and urine volumes, perfusion parameters (intraoperative lactate concentration), postoperative liver function indicators (alanine transaminase [ALT] and aspartate aminotransferase [AST]), postoperative renal function indicators (blood urea nitrogen [BUN] and serum creatinine [Cr]), and postoperative complications.

### Statistical analysis

The predefined primary endpoint was the difference in blood loss between the milrinone group and the nitroglycerin group during the surgery. Compared with the blood loss in patients in the milrinone and nitroglycerin groups undergoing hepatectomy surgery in a previous study, the mean (standard deviation [SD]) blood loss during liver resection was 240.83 (341.50) in the milrinone group and 499.23 (623.86) in the nitroglycerin group [10]. Assuming a type I error of 0.05 and a type II error β of 0.80 and rate of fall off 10%, calculations showed that 34 patients should be included in each group in order to determine a clinically relevant difference (G-power program, two-tailed). Therefore, the total sample size was 68 patients.

Data were analyzed using IBM SPSS statistical software, version 20 (IBM, Chicago, IL, US). Categorical data were presented as numbers (percentages) and compared using the chi-square test or Fisher’s exact tests, as appropriate. Continuous data were presented as the mean (SD) and compared using Student’s *t*-test in the case of homogeneity of variance or presented as the median (interquartile range) and compared using the Mann–Whitney U test in the case of heterogeneity of variance. Repeated-measures analysis of variance was used for intragroup comparisons at different time points. The Kappa test was used to evaluate the consistency of surgical field grading between the surgeon and the first assistant. The Mann–Whitney rank sum test was used to evaluate the difference in surgical visual field grading between the two groups. A *P*-value of < 0.05 was considered statistically significant for all analyses.

## Results

**Fig. 1:**
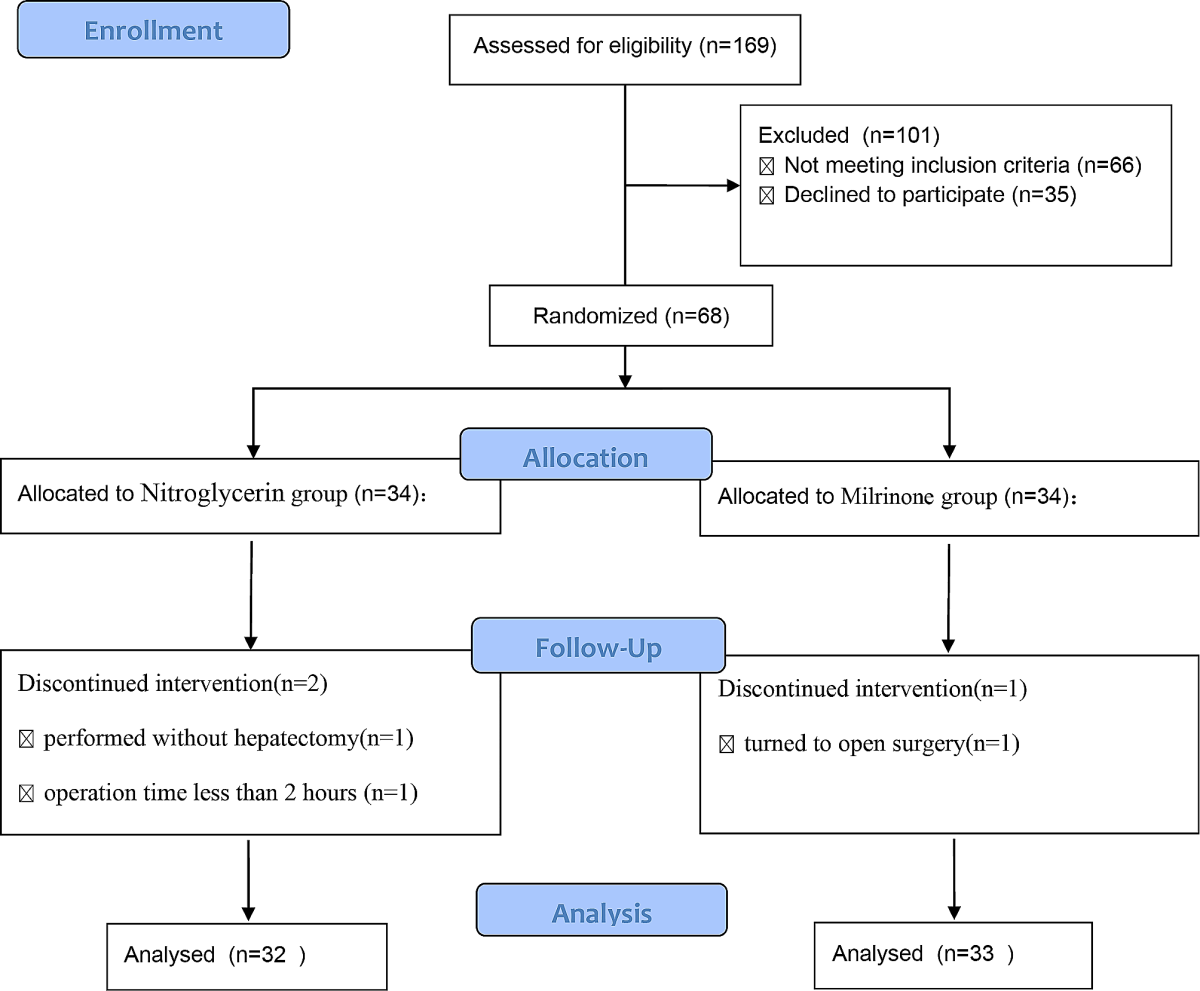
The CONSORT flow diagram

All patients were screened for eligibility, and the data of those enrolled in the present study were recorded consecutively. Out of 169 patients assessed for eligibility, 68 were enrolled and randomized into the two groups. Among these 68 patients, two patients in the nitroglycerin group were excluded from the study. One patient did not undergo hepatectomy because the intraoperative investigation revealed atrophy of the right liver; thus, choledochoscopic lithotomy was performed instead of hepatectomy. Another patient was excluded because of the time required for the surgery was less than 2 h. One patient in the milrinone group was excluded because of unexpected difficulties regarding the surgical procedure and intraoperative bleeding of > 1500 mL; thus, the patient underwent open surgery instead. Therefore, 65 patients (95.6%) completed the study. The CONSORT flow diagram is shown in Fig. 1.

The characteristics of the patients in the two groups did not differ significantly (Table 1). The comparison of the two groups in terms of surgical characteristics and anesthesia characteristics is presented in Table 2. Significant differences were found in fluids administered during hepatectomy, urine volume during hepatectomy, total urine volume, blood loss during hepatectomy, total blood loss, and norepinephrine dosage used in the surgery between the two groups. Blood loss during hepatectomy was less in the milrinone group than in the nitroglycerin group (130.00 [70.00, 260.00] vs. 195.00 [150.00, 260.00] mL, *P* < 0.05), which was similar to the values for total blood loss during the surgery (220.00 [120.00, 380.00] vs. 310.00 [230.00, 380.00] mL, *P* < 0.05). Additionally, a decrease in hemoglobin detected during the routine blood examination before and after the surgery was higher in the nitroglycerin group than in the milrinone group (11.66 ± 6.80 vs. 9.70 ± 9.66 g/L); however, the difference was not statistically significant. Similarly, the decrease in blood platelet count was not significantly different between the two groups. The serum lactate level detected by a blood gas analysis increased after the surgery in both groups; the increase was lower in the milrinone group than in the nitroglycerin group (1.39 ± 0.56 vs. 0.94 ± 0.54, *P* < 0.05).

**Table 1:** Patients characteristics

	Nitroglycerin (*n* = 32)	Milrinone (*n* = 33)	*P* vaule
Sex (N, %) Male Female	22 (68.8%) 10 (31.2%)	22 (66.7%) 11 (33.3%)	1
BMI (kg/m^2^)	23.17 ± 3.48	23.24 ± 3.24	0.933
Age (years)	56.47 ± 8.30	57.64 ± 9.05	0.590
ASA (N, %) I II	4 (12.5%) 28 (87.5%)	5 (15.2%) 28 (84.8%)	1
Comorbidities (N, %) Hypertension Diabetes Hepatitis B	10 (31.2%) 4 (12.5%) 17 (53.1%)	13 (39.4%) 6 (18.2%) 15 (45.5%)	0.669 0.771 0.536

Data were presented as the mean ± SD and N (%). *BMI* body mass index, *ASA* American Society of Anesthesiologists

**Table 2:** Surgical and anesthesia characteristics

	Nitroglycerin (*n* = 32)	Milrinone (*n* = 33)	*P* value
Duration			
Anesthesia (min)	267.50 [198.25; 310.25]	249.00 [98.00; 324.00]	0.768
Surgery (min)	232.00 [149.00; 264.75]	208.00 [151.00; 290.00]	0.896
Hepatectomy (min)	58.50 [41.25; 94.00]	51.00 [35.00; 89.50]	0.348
Pringle maneuver, n (%)	25 (78.13%)	27 (81.82%)	0.710
Inflow occlusion time (min)	40 [15.00; 64.00]	40 [17.50; 61.00]	0.942
Malignancy, n (%)	25(78.13%)	22(66.67%)	0.302
Major liver resection, n (%)	14(43.75%)	12(35.29%)	0.543
Fluid administered (mL)			
Before hepatectomy (mL)	280.00 [200.00; 380.00]	300.00 [235.00; 355.00]	0.577
During hepatectomy (mL)	180.00 [111.25; 325.00]	340.00 [205.00; 405.00]	0.001
Total (mL)	1220.00 [1077.50; 1705.00]	1510.00 [1155.00; 1962.50]	0.172
Urine (mL)			0.993
Before hepatectomy (mL)	60.00 [42.50; 100.00]	90.00 [50.00; 110.00]	0.166
During hepatectomy (mL)	70.00 [40.00; 90.00]	100.00 [70.00; 140.00]	0.002
Total (mL)	250.00 [192.50; 350.00]	300.00 [250.00; 475.00]	0.041
Bleeding (mL)			0.753
Before hepatectomy (mL)	30.00 [25.00; 40.00]	25.00 [20.00; 40.00]	0.203
During hepatectomy (mL)	195.00 [150.00; 260.00]	130.00 [70.00; 260.00]	0.019
Total (mL)	310.00 [230.00; 380.00]	220.00 [120.00; 380.00]	0.031
Hypotension	20(62.50%)	16(48.48%)	0.256
Norepinephrine(µg)	980.00 [610.00; 1310.00]	480.00 [300.00; 760.00]	0.001
Blood transfusion, n (%)	4(12.50%)	5(15.15%)	1.000

Values are expressed as mean ± SD, median (25th,75th) or number (%). Major liver resection: 3 liver segments resected

Surgical field grading during liver resection was significantly better in the milrinone group compared with that in the nitroglycerin group (*P* < 0.05) (Table 3). Moreover, a kappa statistic of 0.727 (95% confidence interval: 0.547–0.907, *P* < 0.001) revealed substantial agreement between the two surgeons.

**Table 3:** Grade of surgical field

Grade	I (sur/assi)	II (sur/assi)	III (sur/assi)	IV (sur/assi)
Nitroglycerin group (*n* = 32)	42(23/19)	21(9/12)	1(0/1)	0(0/0)
Milrinone group (*n* = 33)*	54(28/26)	12(5/7)	0(0/0)	0(0/0)

Values are expressed as numbers. sur/assi: The attending surgeon and the first assistant

Each patient was graded by the attending physician and first assistant independently

Interobserver variability: kappa statistic = 0.727 (95% CI: 0.547–0.907, *P* < 0.001)

*p < 0.05 compared to Nitroglycerin group

**Fig. 2:**
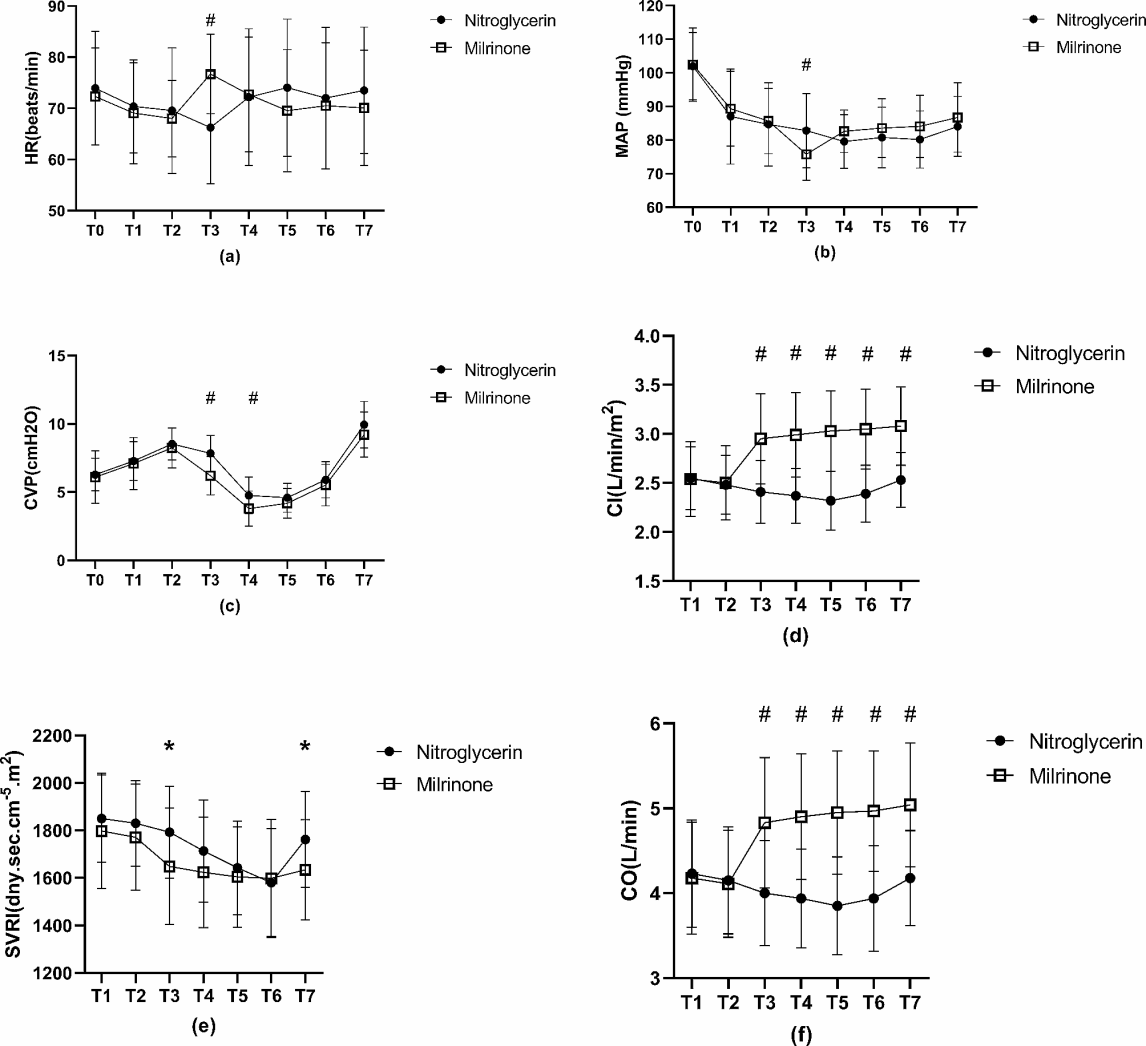
Hemodynamic parameters of two groups during operation (1) #*P* < 0.05 compared to nitroglycerin group; (2) T0: before induction; T1: after induction; T2: before milrinone/nitroglycerin administration; T3: 10 min after milrinone/nitroglycerin administration; T4: before the initiation of liver parenchymal transection; T5: midway through liver parenchymal transection; T6: after the completion of parenchymal transection; T7: at the end of the surgery

The intraoperative hemodynamic parameters in both groups are presented in Fig. 2. The mean HR was greater in the milrinone group than in the nitroglycerin group (T3, Fig. 2, a), whereas the mean MAP was lower in the milrinone group than in the nitroglycerin group 10 min after milrinone/nitroglycerin administration (T3, Fig. 2, b). The mean CVP was lower in the milrinone group than in the nitroglycerin group before the initiation of liver parenchymal transection (T3 and T4, Fig. 2, c). Nevertheless, no statistical differences were observed for HR, MAP, and CVP at the other time points. CI and CO were greater in the milrinone group than in the nitroglycerin group from 10 min after milrinone/nitroglycerin administration to the end of the surgery (T3 to T7, Fig. 2, d and f). SVRI was lower in the milrinone group than in the nitroglycerin group 10 min after milrinone/nitroglycerin administration and at the end of the surgery (T3 and T7, Fig. 2, e).

**Fig. 3:**
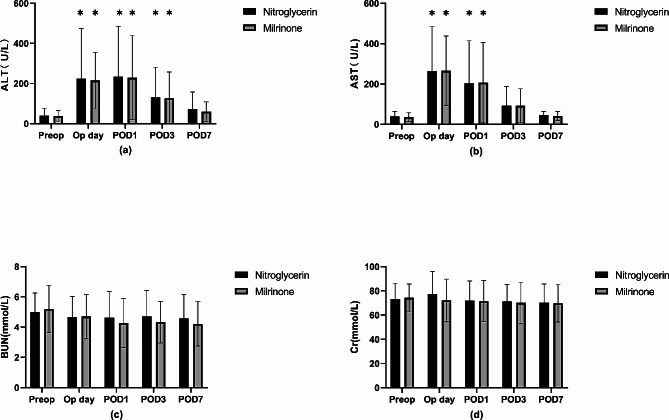
Liver and renal function of two groups (1) #*P* < 0.05 compared to nitroglycerin group; (2) D0: before operation; D1: Postoperative day-1; D3: Postoperative day-3; D7: Postoperative day-7

The mean ALT and AST levels did not differ significantly between the two groups throughout the evaluation period, and both groups showed a similar trend of ALT and AST level alterations (Fig. 3, a and b). Similarly, the mean BUN and Cr levels did not differ significantly between the two groups throughout the evaluation period, and the value was within the normal range throughout the time (Fig. 3, c and d). The incidence of transiently increased urea nitrogen levels was higher in the nitroglycerin group than in the milrinone group after the surgery; however, the difference was not statistically significant (12.50% vs. 6.06%, *P* > 0.05). Additionally, the incidence of postoperative surgical complications, including wound infection, intra-abdominal infection, biliary fistula, postoperative hemorrhage, and liver failure, was similar between the two groups. Next, we classified these postoperative complications according to the Clavien–Dindo classification [13], and the incidence of grade III and higher complications did not differ significantly between the two groups (*P* > 0.05 for both, Table 4).

**Table 4:** Postoperative recovery of two groups

	Nitroglycerin (*n* = 32)	Milrinone (*n* = 33)	*p*-value
Surgical complications	5	2	0.399
wound infection	1	0	0.492
Intra-abdominal infection	1	2	1.000
Biliary fistula	1	0	0.492
Postoperative Hemorrhage	1	0	0.492
Liver failure	1	0	0.492
With complication of Clavien-Dindo grade IIIA or above	2	0	0.238
Hospital mortality	1	0	0.492

Values are expressed as numbers

## Discussion

This is the first randomized clinical trial that investigated and compared the effects of milrinone and nitroglycerin for controlling LCVP on clinical outcomes in patients undergoing LH. Milrinone effectively maintained LCVP during LH. Compared with the nitroglycerin group, the milrinone group showed an equivalent CVP value and more fluids administered during hepatectomy, less blood loss with better surgical field grading, and more stable hemodynamic manifestation with less norepinephrine dosage (*P* > 0.05). Conversely, the two groups showed similar incidences of perioperative complications.

Accumulating evidence has proven that low CVP helps effectively reduce blood loss during hepatectomy (including open hepatectomy and LH) [5, 7, 14]. Generally, inflow occlusion (Pringle maneuver) is the most commonly used clinical technique to reduce blood loss during hepatectomy. After blocking the inflow into the liver, the main source of bleeding is the backflow from a hepatic vein, which is a major problem during liver resections because such substantial backflow bleeding makes it difficult to dissect tissues during parenchymal transection and can occasionally result in massive hemorrhage. Additionally, the hepatic vein drains directly back into the inferior vena cava; thus, hepatic venous pressure is positively correlated to the central vena [15]. Low CVP helps reduce inferior vena cava distension, thereby decreasing hepatic venous pressure. Furthermore, additional bleeding from the hepatic vein resulting from liver resection is reduced because of reduced backflow pressure. A CVP of < 5 mmHg helps effectively reduce intraoperative blood loss and blood transfusion during hepatectomy; thus, the LCVP technique is recommended in liver resection surgery to reduce bleeding [4, 6, 16].

Although low-CVP-based strategies have been proven valuable in reducing intraoperative bleeding and lowering transfusion rates during hepatectomy and liver transplantation, no standard protocol has been developed for LCVP management [4, 6, 7]. Conventional anesthesia used to reduce CVP, including fluid restriction, reversing Trendelenburg position, and diuretic and vasodilator (the most commonly used is nitrates) usage, may decrease cardiac preload, leading to insufficient blood perfusion in vital organs, which may counteract the benefits of low blood loss. Therefore, improving the safety and advantage of the LCVP technique has always been our focus.

Milrinone, a non-digitalis positive inotropic drug, enhances myocardial contractility, improves ventricle relaxation, and dilates blood vessels [17], exerting complementary effects on the patients. Milrinone reduced CVP with better hemodynamic manifestation and improved postoperative recovery by an improvement shown compared with nitroglycerin during open hepatectomy [9, 10]. The present study confirmed the effectiveness of milrinone in maintaining LCVP during LH. Additionally, the milrinone group showed less blood loss and better surgical field grading than did the nitroglycerin group (*P* < 0.05). The probable underlying mechanism is that the vasodilating effect of milrinone reduces cardiac preload and CVP, while enhanced lusitropy may also augment venous drainage, increasing venous return from the inferior vena cava and hepatic vein. These events led to less hepatic congestion, less backflow bleeding in the operating field, and a dry surgical field. The dry surgical field helped in dissection during parenchymal transection, facilitated the surgery, and lowered bleeding during the surgery.

The standards for CVP control in the literature have evolved with advancements in related research. The earliest studies on LCVP during liver resection used target values of 5–10 mmHg [18]. Subsequently, multiple studies used target values of 3–6 mmHg [6, 16, 19]. The target CVP was usually set at 5 mmHg or less before and during liver parenchymal transection and was the only hemodynamic goal of LCVP management in almost all later studies [10, 11]. Maintaining LCVP at a certain value helps obtain a good surgical field. Surgeons prefer even lower CVP, which would help obtain a better surgical field. However, extremely low CVP may be related to complications, especially in elderly individuals and people with heart or cerebral diseases [20, 21]. In our opinion, the target value of intraoperative CVP should be dynamic and individualized. After all, improvement in surgical techniques have played a major role in reducing bleeding. Therefore, LCVP management should not be conventional but should be based on a patient’s basic condition, intraoperative bleeding risk, and surgical field grading. Moreover, anesthesiologists should provide a good surgical field and communicate promptly with surgeons, as well as achieve optimal CVP and ensure tissue perfusion. Herein, the fluid infusion rate was adjusted by the attending anesthesiologist per the CVP and surgical field grading during liver parenchymal transection. When the surgical field grading was at grade 2 or less, CVP was appropriately increased to ensure tissue perfusion. The volume of fluids administered to the milrinone group during liver parenchymal transection was significantly more than that administered to the nitroglycerin group. However, the volume of fluids administered before liver parenchymal transection and total fluid infusion did not differ between the two groups. More volume of fluids administered during liver parenchymal transection in milrinone-induced low CVP was probably attributed to the presence of a relatively better surgical field. To the best of our knowledge, few studies have focused on the issue of dynamic and individualized LCVP management and the possible effects of using different methods on patient outcomes. This is a strength of the present study, and more studies should be performed on dynamic and individualized CVP management.

Irrespective of undetermined clinical significance, the present study showed that compared with patients in the nitroglycerin group, patients in the milrinone group probably exhibited improved tissue perfusion as indicated by lower serum lactate levels, higher urine output, and lower need for vasopressor support. Additionally, the incidence of transiently increased urea nitrogen was higher in the nitroglycerin group than in the milrinone group after the surgery; however, the difference was not statistically significant (12.50% vs. 6.06%, *P* > 0.05). There are three possible explanations for this phenomenon. First, several studies showed that milrinone increased CO and CI because of inotropic actions while simultaneously exhibiting low systemic vascular resistance owing to a potent vasodilator effect during liver resection [9, 10]. This effect ensured adequate end-organ perfusion and was affirmed by the present study. Conversely, nitroglycerin decreased CO and blood pressure [22]. Second, studies on induced hypotension reported that milrinone exerted beneficial effects on renal and cerebral perfusion, suggesting the advantageous effect of milrinone on end organs [23–25]. Third, although no difference was observed in total fluid infusion between the two groups, more volume of fluids was administered during liver parenchymal transection in milrinone-induced low CVP, and this increase in tissue perfusion probably improved tissue metabolism during hepatectomy.

The optimal dose of milrinone for reducing CVP and bleeding from the surgical field of the incised liver surface during LH remains unclear. A high loading dose (25–50 µg/kg) always leads to transient hypotension. To avoid hypotension caused by rapid milrinone administration, Yang P et al. skipped the loading dose and infused milrinone at a rate of 0.5 µg/kg/min from the beginning of the surgery; consequently, the CVP decreased gradually and reached the expected value by the time of liver parenchymal transection [10]. A previous study showed that 150 min after milrinone infusion at a rate of 0.5 µg/kg/min without a loading dose (in a 50-kg patient) resulted in a milrinone plasma concentration of 58 µg/L, which could be the target plasma concentration to achieve systemic vasodilation using milrinone during donor hepatectomy [12, 26]. Herein, initially, milrinone was administered at a low loading dose of 10 µg/kg over 10 min at the beginning of the surgery, followed by infusion at a rate of 0.2–0.5 µg/kg/min until the removal of the liver lesions. The mean HR was greater, and the mean MAP and CVP were lower in the milrinone group than in the nitroglycerin group after administering the milrinone loading dose (*P* < 0.05, Fig. 2). Although blood pressure decreased, this decrease was in the normal clinical range; thus, no intervention was needed for hypotension. Hence, we thought that the milrinone dosage used in the present study was appropriate to exert a prompt low CVP effect without undesirable hypotension. A similar result has been reported by Ryu et al. [9]. To the best of our knowledge, studies examining the optimal dose of milrinone for reducing CVP in patients undergoing LH are lacking. Therefore, more studies should be performed to address this important issue.

Nevertheless, three limitations of the present study should be considered. First, the study was performed at a single center, and the sample size was decided to detect only the difference in blood loss between the groups, which was the primary outcome of the study. Second, although we only discovered transiently mild hypotension after a low intravenous loading dose, a few adverse effects of milrinone have been reported. Thus, caution should be exercised when extrapolating its administration dose to a broader spectrum of patients undergoing LH, such as elderly individuals and those with congestive heart failure because unsustained ventricular tachyarrhythmia has been reported in such patients [27]. Third, although we followed a hemodynamic management protocol, the volume of fluids administered during liver parenchymal transection was different between the groups, which probably contributed to variations in the tissue perfusion index and led to some bias.

## Conclusion

Conventional anesthesia used to reduce CVP may decrease blood perfusion in vital organs, which may counteract the benefits of low blood loss. Herein, we investigated different pharmacological interventions to maximize the benefits of LCVP in LH. We showed that compared with the intraoperative infusion of nitroglycerin, the intraoperative infusion of milrinone maintained LCVP and hemodynamic stability during LH while reducing intraoperative blood loss and providing a better surgical field.

## Data Availability

The data used and/or analyzed during the current study are available from the corresponding author upon reasonable request.

## Abbreviations

LH: Laparoscopic hepatectomy
LCVP: low central venous pressure
ERAS: Enhanced Recovery After Surgery
IV: intravenous
MAP: mean arterial pressure
ASA: American Society of Anesthesiologists
BMI: body mass index
CI: cardiac index
CO: cardiac output
SVRI: systemic vascular resistance index
PetCO_2_: end–tidal carbon dioxide partial pressure
BIS: bispectral index score
HR: heart rate
SpO_2_: pulse oximetry
ALT: alanine transaminase
AST: aspartate aminotransferase
BUN: blood urea nitrogen
Cr: serum creatinine
